# 93137 Interrogating cardio-protective MTSS1 variants in human populations

**DOI:** 10.1017/cts.2021.718

**Published:** 2021-03-30

**Authors:** Megan F. Burke, Michael Morley, Yifan Yang, Theodore Drivas, Mingyao Li, Mingyao Ritchie, Thomas Cappola

**Affiliations:** Perelman School of Medicine, University of Pennsylvania

## Abstract

ABSTRACT IMPACT: It is our hope that a better understanding of the relationship between genetic variants that influence heart failure precursor traits will not only inform clinical care, but enable better assessment of inherited risk and will identify new biological targets for drug development. OBJECTIVES/GOALS: In this project, using a large-scale human genomic dataset with extensive phenotype data available, we intend to interrogate the known MTSS1 variants that have been associated with heart failure (HF) in previous GWAS studies in order to understand the directionality and mechanisms of their effects. METHODS/STUDY POPULATION: Data was obtained from the UK Biobank, a large prospective cohort of ˜500,000 patients across the United Kingdom with extensive phenotype data, including ˜50,000 patients with cardiac MRI and ˜200,000 with whole exome sequencing. We test for associations between genetic variants at the MTSS1 locus and HF precursor traits using logistic regression or linear regression, adjusting for age, gender, and principal components (PCs) of ancestry. For rare variant analyses we ‘bin’ rare variants (MAF < 0.01) using the software tool BioBin to aggregate low frequency genetic variants into single genetic units. RESULTS/ANTICIPATED RESULTS: Preliminary data have shown that variants in the known MTSS1 enhancer region which reduce MTSS1 expression are associated with smaller, more contractile hearts. We anticipate that common variants known to reduce enhancer activity will attenuate heart failure precursor traits, will be associated with a reduced risk clinical heart failure, and will favorably impact clinical outcomes once HF is established. We also anticipate that rare exonic variants predicted to impair MTSS1 function will attenuate heart failure precursor traits. DISCUSSION/SIGNIFICANCE OF FINDINGS: Through this work, we intend to take advantage of multiple novel approaches to better understand a complex disease process, identify a new potential therapeutic target (namely one that targets cardiac function), and to determine which patient subgroups will benefit from this our therapeutic interventions and why.

